# Benevolent paternalism and player transition in Fiji soccer: insights from the Global South

**DOI:** 10.3389/fspor.2026.1678889

**Published:** 2026-01-27

**Authors:** Kieran E. James, Henry D. Tuidraki, Sheikh A. Tanzil

**Affiliations:** 1 School of Business and Creative Industries, University of the West of Scotland, Paisley, United Kingdom; 2 Independent Researcher, Nadi, Fiji; 3 School of Accounting, Finance and Economics (SAFE), The University of the South Pacific, Suva, Fiji

**Keywords:** athlete transitions into retirement, benevolent paternalism, cultural hegemony, Fiji Indians, Fiji islands, Fiji soccer, indigenous Fijians

## Abstract

**Introduction:**

Because Fiji is a relatively poor and remote Global South developing country, it is plagued by poor infrastructure, financial problems, and a lack of trained experts who also can relate to athletes in a culturally appropriate manner. In this article, we compare Global North coaching practices with traditional practices in Fiji, which are based mostly on benevolent paternalism and cultural hegemony. A specific focus is the transition of athletes into retirement.

**Methods:**

Case studies of Nadi Soccer Association and Ba Football Association are utilized based on ethnographic data obtained from seven interviews with ex-players, one interview with an ex-administrator, and one interview with an ex-team doctor as well as extended participant-observation.

**Results:**

The Fiji Football Association (FFA) is seen as working hard now to consider the mental and physical welfare of ex-players after a slow start although the primary initiatives have come from ex-player self-help organizations such as Nadi Legends Club.

**Discussion:**

Although better sport coaching and sport psychology can contribute, they need to be conducted in a culturally appropriate way where Indigenous Fijian and Fiji Indian cultures are worked with, and benevolently paternal administrators and coaches are not cast aside or marginalized.

## Introduction

This article explores the style of management in soccer in Fiji and how it impacts a player's transition into retirement. The traditional management style can best be described as a mixture of benevolent paternalism and cultural hegemony. We define “benevolent paternalism” as a “neo-feudal approach where a manager or coach builds up a long-term paternal relation with the player, based on culturally engrained styles of relating, and shows a commitment to the player's welfare of a type that might seem intrusive in Western contexts.” We define cultural hegemony as “a personal, traditional, and culture-based way of relating and mentoring which uses cultural norms and cues extensively to encourage, motivate, monitor, discipline, and control.” It is important to point out that there remains at the level of the national regulatory body a kind of corporate style that does not fit the Global South. The ex-players need funds for survival and to attend social functions, including official functions held for their benefit by the Fiji Football Association (FFA). However, as we will see, the league body assumes that they can make their own way to the venue without financial and logistical assistance. In fact, the league understands the reality of poverty in Fiji as well as anybody but can shut this knowledge out, in a kind of doublethink. Furthermore, the lifestyles of the executives are very different from those of most of the ex-players as they (the executives) operate at a much higher level of income and lifestyle opportunity, almost as a globetrotter class, and can become physically as well as mentally (in the realm of thought) detached from others.

Even in the Global North countries, where professional footballers' salaries have risen to what would have been unimaginable levels even a generation ago, ex-players may suffer serious depression upon retirement (1). While it was not acceptable in the past to expose your own mental health struggles, the situation is changing.

As an example, the former Scottish footballer, Kevin McNaughton (aged 43), who played for Aberdeen, Cardiff City, Inverness Caledonian Thistle, and Forfar Athletic, has reported experiencing severe depression and suicidal thoughts (1). In another case, the son of the former Chelsea and Glasgow Rangers player Ray Wilkins, who died aged only 61, spoke at his late father's funeral about his long-term battles with alcoholism and depression (2). The main causal factors behind postretirement struggles appear to be loss of camaraderie and solidarity, on the one hand, and the absence of an organized daily routine on the other. Also, the subject position [(3), p. 222; (4), pp. 331, 336; (5), pp. 244–245; (6), pp. 56, 65] of footballer no longer applies, people fade from the public eye and are not given the attention or interest that they once enjoyed. Colin Gordon (5) has defined “subject position” within Michel Foucault's (4) philosophical framework of power/knowledge as: “the social and discursive location from which an individual speaks or is positioned within a particular discourse, influencing how they are perceived and understood.” Except for the very few, privileges decline as does the right to be consulted or listened to on important issues, which gets to the core of what Foucault meant by the term “subject position”. In McNaughton's words, cited in Cassidy (1): “It's a fishbowl and you’re just surrounded by love, even just walking along the street. And then suddenly I was just isolated and I struggled, just not being a footballer anymore and having that routine. I was getting up and just doing nothing.”

A depressing example was the case of the Australian Rules footballer, the late David Granger (1955–2024), nicknamed “Grave Danger” by the fans, who played for Port Adelaide in the South Australian National Football League (SANFL). He was used as an aggressive player who king-hit opponents back in an era when play was a lot more violent and unruly than it is today. On one such occasion, he king-hit an opposition player and felt betrayed, later, when coach John Cahill refused to admit that he had assigned Granger the task. After retirement, Granger spiralled into depression and, on one occasion, feeling alienated from his former club, he broke into the changerooms at Port Adelaide's Alberton Oval and attempted suicide next to his old locker. Granger was sentenced to jail in 2003 for cannabis possession (7) and was convicted of assaulting a Member of Parliament in 2015 (8).

Clubs at one time felt no obvious or clear-cut moral responsibility for the wellbeing of ex-players once they left the playing staff list [(9), pp. 102, 106]. Even now legal liability is limited to certain items such as contractual considerations and the treatment of ongoing injuries. Players experience further anxiety nowadays due to awareness of the risk of dementia from repeated heading of the ball during their playing careers (10, 11). In the longer term, clubs' legal liabilities may extend even into this area of long-term injury where cause-and-effects are more difficult to establish without any doubt. Researchers have recommended that the Scottish SNP (Scottish National Party) government ensure that dementia and other conditions associated with the game are classed as industrial injuries (10). Clubs and regulatory bodies need to adopt a considered and well-informed policy approach towards players on the transition to retirement, realizing that it may not be an easy transition for a whole host of reasons.

What may be ideal, even in the Global North, where nowadays players are from a wide variety of cultural and ethnic backgrounds, is a mix of planning, science, expertise, and an empathetic approach that is committed to long-term relationships that don't just end when the player leaves the first team [(9), p. 110]. The benevolently paternal approach of many Fiji club and district association administrators may not be a bad model if balanced out with more modern considerations and better financing. While the transition to retirement in Fiji is typically stark, as it “relegates” the Indigenous player to customary village life, and most ex-players to relative poverty, the effort needed to remain in the life of the ex-player can be an outstanding example of (soccer) community concern. Village life engulfs the player–the player appreciates the customary life and the roles and subject positions they enter. But the transition is a major one as the player becomes removed from public recognition and exaltation [(9), p. 102: (12, 13), p. 60] as the village is only a semi-public realm. The player has subject positions open up within village customary life, whereas subject positions in relation to the sport tend to close as the ability to move into coaching or administration post-retirement is correlated with race/ethnicity (14–18) and job opportunities are few and far between.

This article will look at the self-help organization, the Nadi Legends Club, founded by former Nadi players and administrators, and mention Bobby Tikaram and his efforts to support players and ex-players, which date back to his time as president of Airport Soccer Club in the Nadi district club competition in the 1970s and 1980s. Then we consider difficulties faced by other ex-players in the face of apathy within an overall corporate approach from league or district administrators, looking at the case of Ba and Fiji stars Inia “Golden Boot” Bola and Semi Tabaiwalu. We also note efforts to honour and support ex-players by the FFA in more recent times.

Our work advances the understanding of athlete transition and coaching culture in the Global South by focusing on a small island nation not renowned for its soccer expertise and drawing upon detailed personal and group interviews with a sample of revered ex-soccer stars from the 1980s, a strong era in the history of the local game. Participation-observation allowed the first author to observe relationships, cultural norms, meeting procedures, and the overall running of the game in the modern day amidst poverty and geographic isolation. The next section is about athlete transition in the European or Global North literature. Then we present a background to Fiji soccer section followed by a Methodology section. Then we provide a Findings section on athlete transition in Fiji soccer that draws upon our primary data. We finish with an extended Conclusion that aims to locate and connect divergent data, contexts, and approaches.

## The European literature on athlete transitions into retirement

In his survey-of-the-field chapter in the book *Athlete transitions into retirement*, Andreas Küttel [(19), p. 18], drawing upon Stambulova and Wylleman (20), claims that a professional culture which aims to assist career transitions should be based on the following principles: (1) A *cultural-specific approach* to help athletes adjust within a particular sports system, society, and culture. (2) A *whole-career approach* to help athletes cope with both normative and non-normative transitions throughout the whole course of an athletic career. (3) A *whole-person approach* to help athletes deal with transitions in various spheres of life. (4) A *developmental approach* to help athletes linking their past career experience, present situation, and the plans for their future. (5) An *individual approach* to accommodate the athletes' perceptions of the transition and their distinctive resources and barrier(s) for the transition(s). (6) A *transferable skills approach* to teach athletes life skills that are applicable both in and outside sport and in the athletic and post-athletic career.

Clearly, even at well-resourced sporting clubs in richer countries, some or all these attributes may not be operating at a suitably high level, as Tonge (13) documents in regards English soccer and Agnew and Abery (9) report for Australian Rules football. In poorer Global South countries, we can expect more gaps in policies and programmes, but benevolent paternalism from individual leaders may partly compensate for this, as may self-help organizations set up and run by ex-players. We will see in the Findings section the positive role played by Nadi Legends Club in assisting ex-Nadi Soccer Association players in Fiji.

Tonge [(13), p. 69], writing from the perspective of encouraging athletes going through difficult periods in their playing careers to consult sports psychologists, recommends as follows: (1) To deal with the challenges that crop up, a broader psychological approach may be needed. (2) Many professional football players will already possess high-quality mental skills and be competent with certain approaches that have been taught within the academy (i.e., imagery, concentration, goal setting). Something else would be useful. (3) An existential sport psychology approach can help fill this gap. This approach can help the player consider who they are at a particular moment in time and consider a range of choices, both narrow and broad, to help progress and move forward.

Tonge (13) presents five semi-fictional case studies that each include quotes of discussions between football players and sports psychologists, which are then followed in each case by commentary and interpretation. Some additional points raised by the author are as follows: Case 1: In transitioning out of professional sport, some athletes may have a good experience while others may have a difficult one (21). What is important is the context, especially whether the retirement is chosen or forced [(9), p. 102; (22), pp. 24–25]. The career journey over time can become a key part of a player's identity (23). The more significant the “player” identity or status is to one's total identity the harder the transition will be, other things being equal [(22), p. 25]. In the case of a crippling injury, a player may lose “meaning and purpose” and even become “a shadow of their former selves” [(13), p. 60]. To get honest and complete disclosure by a player to a sports psychologist requires trust and the assurance of confidentiality (24).

Tonge [(13), p. 60] goes on to explain that the transition into retirement is “probably the toughest thing” that people will have to face. He reiterates [(13), p. 60], citing Corlett (25), that “quick fixes and temporary solutions” are to be avoided as they may create more harm than good. Injury problems can impede planning and result in financial hardship. The Professional Footballers' Association (26) has reported that loss in income when leaving the sport can be as much as 70% and many football players go bankrupt within five years of retirement. The player in Case 1 managed to turn his depression into a positive outcome when he began to receive fulfillment from coaching. While a coach can be of assistance, a sports psychologist may be able to help a player with identity issues. The psychologist can investigate and map out potential pathways forwards with the player.

In Case 2, the player was loaned out from a Premier League club to a League One (third tier) club, a process which can cause anxiety (23). Corlett (27) suggests that facing the situation and making a considered decision or set of decisions takes courage and Tonge's (13) existentialist understanding of the athlete's dilemmas and, by implication, the sports psychologist's role is evident here. Tonge [(13), p. 63] echoes Frankl (28) by saying that the sports psychologist should guide and accompany the player through the choice process and point out that options will always be available even in seemingly deadlocked situations. Buber (29) refers to *stunted person centre* which is the concept that by ignoring troubles one becomes less able to deal with future problems as the habit of not being decisive sets in.

In Case 3, a player is being criticized by the manager and fans, including fans on social media. The existentialist approach from the sports psychologist is again evident in the quoted conversation and discussion. The psychologist reiterated in this case that one's footballer identity should be only one part of one's total identity. Tonge [(13), p. 64] explains that it can be difficult to get into a discussion along these lines at a football club. What is needed is someone that the player can open up to, a person who is trusted, and a person who can explore with the player issues of “identity, anxiety and transitions” [(13), p. 65]. It helps if the psychologist has played the sport too and has a feel for it.

Agnew and Abery (9) look at the topic of players retiring from the national Australian Football League (AFL) competition and dropping a level to the state-based South Australian National Football League (SANFL). Clubs in the SANFL are only semi-professional, are lacking resources compared to AFL clubs, and attract crowds of only one to two thousand. These authors claim that dropping back to SANFL level before completing retirement has several advantages as club networks can help a new player find a job outside the game; there is an opportunity to mentor younger players; and the chance exists to be redrafted to the AFL. However, players must have realistic expectations of what clubs can do for them at this level due to lack of money and paid staff. It could also be that retirement problems are just delayed a few years rather than finished with. An important distinction made explicit by those authors is between players delisted by the AFL club (forced removal) and those that retire voluntarily from the top-level competition. In between those categories are countless cases of pressure being applied to a player to leave and the player in the end acceding to the pressure while officially leaving “voluntarily” (e.g., Connor Goldson at Glasgow Rangers in 2024).

## Background

Fiji soccer is a conundrum and an enigma, a sport largely unknown overseas but deeply and passionately supported by a loyal band of mostly Fiji Indian men (and some women) now resident either in Fiji or in the Global North countries of Australia, Canada, New Zealand, and the USA.^1^ As James and Nadan (14–18) report, the émigré communities have taken their love for the competition of their homeland to an extent rivaling the Croatian émigrés, with competitions and teams featuring the names of their equivalents in Fiji in the countries mentioned above, so we have teams known as Sydney Rewa and Vancouver Nadi. Even the lesser Fiji tournaments organized by religious communities, such as Sangam (Hindu) and Muslim, and ethnicities such as Gujarati have their equivalents in some of the Diaspora cities. We now get the phenomenon of “soccer tourists” who return to the islands both to visit family and attend soccer games–these trips give them the opportunity to reconnect with both memories and old friends (15, 17). It would be completely wrong to question the sincerity of the émigrés. It is important to note that their fandom does have the effect of marking them out, in their own eyes, as distinct from other Indians and Pakistanis in the West.

The first clubs and leagues, excluding those games between European teams and visiting ship crewmen, typical of the colonial-era, emerged in the southeast of the main island Viti Levu in the 1920s [(30), pp. 8–14]. In the first half of the twentieth century, there were European leagues, Indian leagues (which admitted Chinese players), and “native” leagues for the Indigenous Fijians. The earliest documented Fiji Indian clubs were Sunshine Sports (from Suva) and Sitare Hind (from Rewa) who were in existence as early as 1922. A Northern Natives League for Indigenous players, based in the Western Fiji towns around Ba and Rakiraki, began playing about 1927 [(30), p. 11].

The first annual knockout Inter-district Championship (IDC) tournament, contested between regional association or district teams, as opposed to club teams, was held in 1938 [(30), pp. 17–19]. Apart from the military coup year, 1987, the IDC has been contested ever since, making it one of the world's oldest continually running sporting tournaments. The IDC was joined by the Battle of the Giants (BOG) (established 1978) and Fiji FACT (established 1991). As the national league of two divisions was set up in 1977, there are four annual trophies competed for by district teams every season.

Another significant event was the opening of the Indian league to Indigenous players for the beginning of the 1962 season with Indigenous star Esala Masi Senior being a key recruit [(30), pp. 39–40, 42]. The Indian FA dropped the word “Indian” from its name and effectively at that point rewrote its own history as national-league history [(30), p. 39]. In the twilight years of the segregated leagues, the Namoli Tigers won the Northern Native League title for an astonishing nine seasons in a row.

Soccer, because it derives the bulk of its support from the Fiji Indian community, has tended to historically be strongest in the sugarcane belts of Western Fiji on the main island Viti Levu and around Labasa on the second island Vanua Levu. While Lautoka Blues, based in the Western Fiji city of Lautoka, was the most powerful team in the 1950s and 1960s, by the 1970s the crown had moved 30 kilometres northeast to Ba, a small market town and manufacturing and distribution centre (30). As Ba has always had a Fiji Indian majority, this explains to some extent Ba's continued success as it has been financially and morally supported by the various Fiji Indian-owned Small and Medium Enterprises (SMEs) based in the town. It is the only town or city in Fiji where soccer is more popular than rugby and, anecdotally, is regarded as the only town or city where people discuss soccer 24-hours-a-day and 7-days-a-week rather than just on match days. It hosts the famous Soccer Ball Café on the main street where a huge soccer ball has pride of place on the roadside and in fact houses the restaurant's kitchen. While Ba won a record-breaking seven IDCs in a row from 1975 to 1980, Nadi's four league titles in a row from 1980 to 1983 are seen as an equally impressive feat [(30), pp. 60–99].

A significant landmark event, that occurred on a dark road late one night in August 1984, and not on a football field, haunts the history of Fiji soccer and especially in the cane belt [(30), p. 103]. On the way home from a nurses' dance in the neighbouring town of Tavua, a utility truck containing three key Ba players smashed into the pulley of a parked sugarcane truck which had stopped in front of a convenience store. Although it was on the main highway, lighting was poor and the truck and its surrounds were shrouded in darkness that prevented the hired driver from slamming on his brakes or swerving in time. Ba and Fiji captain Josiah “Joe” Tubuna died immediately as he was sitting in the middle front seat (he was hit by the truck's pulley) while Inia “Golden Boot” Bola suffered life-changing mental and physical injuries. The third soccer icon, Semi Tabaiwalu, did not escape unscathed. A public funeral for Tubuna, held at Ba's Govind Park ground, was attended by five thousand people of all ages and ethnicities. Bola played only one more half-season for Ba. Facing a relegation playoff at the end of the 1985 season, he was recalled to the Ba team for one last effort in the striker position. Ba easily accounted for Tailevu-Naitasari 7–1, with Bola scoring three or four goals (sources vary with Prasad writing three and Bola in interview stating four) [(30), p. 108].^2^ In typical pragmatic Fiji style, both team managements agreed to forget about the second leg. By 1986 Ba was up at the top again, chasing and securing trophies.

Although cash payments to players were minimal in the 1980s, Tubuna was widely seen as a new type of Indigenous player–assertive, one who knew his rights, and one who sold his services to the highest bidder. Like Augustin Thoman in the 1950s and 1960s, he was recruited by Ba although he hailed from Lautoka. He was admired by the Indigenous villagers for his assertiveness and forthrightness, while Fiji Indian soccer fans respected his on-field courage and leadership skills.

In May 1985 Newcastle United came over from England to Fiji to play two friendly games against the Fiji national team. The first game, played in sweltering humidity on a Saturday afternoon at Nadi's Prince Charles Park, was won 3-0 by Fiji, while Newcastle won the second game 2-0 on the Tuesday night in Suva [(14, 30), p. 104]. It was a remarkable performance by Fiji, although the national team was at a historic peak in the 1980s, culminating in a 1-0 win over Australia in 1988 also at Prince Charles Park.

## Materials and methods

The first author arrived in Lautoka in May 2013 to take up an academic appointment at the local university. During the 2013 season, I (first author) became a regular attendee at the home games of Lautoka Blues in the Fiji Premier League. I enjoyed following and learning about the league but was limited to newspapers, books [such as (30, 31)], and personal observation at games. Around December 2013 I met by chance Henry Dyer (name changed) at the Deep Sea (Indigenous) Pub (now closed) on the Nadi main street. During our long conversation, he persuaded me that he was a former Fiji national-league and national team player and people I knew and we both met in coming days were able to confirm it. Plus, his fitness was still at a high-level judging by his walking speed. We agreed, after I suggested it, that we would co-write his memoirs and I could use the information and opinions collected for journal articles. We met on 20 Thursday afternoons (average meeting time: 3 h) at various Nadi Town Centre venues to write the book. These sessions lasted from May 2014 to April 2015.

Early on I was introduced by Dyer to his long-term mentor and friend, Mr Bobby Tikaram, a former Airport Soccer Club (ASC) president and Nadi Soccer Association vice-president. Relevant to this article, I was told by Tikaram and Dyer about how Tikaram noticed Dyer as a 15-year-old at a secondary schools' soccer tournament but resisted approaching him as he was then living in Lautoka. About three years later, in 1981, Dyer was 18 years old and Tikaram spotted him again, playing touch rugby at the Airport grounds. Both men renewed their old acquaintance, and Tikaram was able to recruit Dyer for the ASC now that he (Dyer) was living in Nadi. At the time of his recruitment, Tikaram gifted him a pair of soccer boots consistent with our earlier comments about benevolent paternal Fiji Indian administrators in the sport. Shortly afterwards, Dyer was selected to play for the Nadi Soccer Association district team in what is now the Fiji Premier League where he received immediate attention as a young star on the block. Consistent with ethnography, I spent countless hours interacting and socializing with Dyer and his friends and family members at Nadi Town Centre, Lautoka City Centre, Namoli Village (Lautoka), and Nakavu Village (Nadi). At that time Dyer was serving as assistant village headman at Nakavu Village and now serves as elected village headman. We would often meet ex-players around town in Lautoka and Nadi and in the Indigenous villages. During 2014–2015, the late Lautoka defender, Wally Mausio, for example, was a regular afternoon drinker at the Lautoka Club. I was always observing and studying interactions and relationships and would often ask Dyer or someone else present about why things were happening and what events meant within their specific cultural context.

The second stage of the project, conducted between June and October 2015, involved interviewing other ex-soccer stars from the Western Fiji region who had played during Dyer's era. We planned to interview as many players as possible who had played in the iconic 1982 IDC Final between Nadi and Ba when bad light had stopped play during the middle of the penalty shootout (17). We, meaning Dyer and me, since Dyer had now become a co-researcher and co-interviewer, also wanted to interview both Indigenous Fijian and Fiji Indian ex-players to obtain a more balanced perspective on issues pertaining to race/ethnicity and their relations with administrative and managerial control. We ended up interviewing 4 ex-Ba players and 1 ex-Nadi player from that match, which, if we include Dyer as well, amounts to 27% of the starting lineups (6/22). Interviews lasted between 1 and 3 h and players' wives participated in some of the interviews.

The third and last stage of data collection involved me travelling four hours by public bus from Lautoka to Suva in November 2015 to access hard copies of newspapers at the *Fiji Times* head office. As my time and money were both limited, I chose to take notes centering on four key events as follows: (a) the 1982 IDC Final; (b) the 1984 death-and-funeral of Tubuna; (c) the 1985 Fiji-vs.-Newcastle United games; and (d) the 1988 Fiji-vs.-Australia matches.

After each session with Dyer, he would visit the University and read the typed-up transcript from our previous session, He would then ask for changes when necessary and finally give his approval The transcripts were posted on our online soccer blog. In my analysis, I performed triangulation between Dyer's comments and verified facts from history books or newspaper sources. When facts or details were in conflict, newspaper sources were given primacy on Dyer's instructions. I analyzed the data and separated it according to emergent themes. I highlighted key sections in my notebooks according to emergent themes. Special attention was paid to issues of race/class, cultural hegemony, and benevolent paternalism, and Foucault's power/knowledge framework emerged as a useful and insightful tool for interpretation. We define cultural hegemony as “a personal, traditional, and culture-based way of relating and mentoring which uses cultural norms and cues extensively to encourage, motivate, monitor, discipline, and control.”

Validity and reliability are important goals in qualitative research. They are difficult to guarantee or ensure in ethnography such as in this project where it was difficult to gain access to interviewees due to time and money constraints and the fact that many ex-players have died or emigrated to Western countries. We aimed to access people from a mixture of teams and ethnic groups and made sure relevant questions were asked of each person whether in formal interviews or conversations. Due to time and money constraints, we stuck to researching Western Fiji not the whole country. I attended matches and social functions including the 2014 FFA Veterans' Dinner. I was respected because I was working at the local University as professor and this too opened doors. I was a white Australian man in my mid-forties, and this placed me well as I was not seen to be aligned with any ethnic group or religion. However, I had to convince Fiji Indians that I was not biased against them due to my close friendship with Dyer who is of mixed Indigenous Fijian and white British descent. All interactions had to be marked with decorum and politeness and respect but being a soccer fan meant that I showed genuine interest in the game. I adhered to village rules and logic while in village space such as showing decorum, not approaching anyone without a valid purpose, and going straight to and from my destination without wandering about. I was welcome on village land largely because of my close association with Dyer and his family. This study could be replicated, given details we have provided, but it may be hard to access the same participants as we accessed. For example, Julie Sami passed away in October 2025.

## Findings

### Introduction to Dyer and Tikaram

As we have already mentioned, in 1981, Tikaram, then the ASC president, spotted Dyer playing touch rugby at the airport grounds in Namaka. By this time, he (Dyer) was living in the Nadi area and Tikaram was able to sign him for ASC. In benevolently paternal style, Tikaram bought Dyer a pair of soccer boots. They have a long-term friendship based on trust and mutual respect which lasts up until the present day. Tikaram's memory was faulty as the two reminisced about Dyer's first senior club game for ASC, held at the Nadi Sangam School grounds. This game, against Blues Soccer Club, was won 2-0 by ASC with Dyer scoring one goal and the late Emasi “Bacardi” Koroi scoring the other. As Western Fiji is a money-poor but time-rich society, long-term relations based on trust are a key ingredient of Fiji soccer management and coaching and should not be looked down upon by residents of Global North countries where more resources and better education might deem such approaches “outdated” or “unprofessional”.

### Dyer's playing career highlights

Now we fast-forward several years because the topic of this article is player transitions into retirement. But first we give a brief rundown of the main aspects of Dyer's playing career. He commenced his career with ASC and the Nadi district team in 1981 and by 1983 was a member of the Fiji national team. Dyer played for Nadi in the legendary 1982 IDC Final between Nadi and Ba when bad light stopped play during the penalty shootout. He was in the Fiji team which beat Newcastle United 3-0 in a friendly match at Prince Charles Park, Nadi, in May 1985 and played in the second game held in Suva, won 2-0 by Newcastle. He switched to Lautoka Blues, in an outstanding era for the team, 1984–1986, but arrived too late to fully participate in their trophy successes.^3^ During that time he played in one of the Blues' best-ever teams which featured Sam Lal, Niko Lilo, Wally Mausio, John Monday, Kelemedi “Cheetah” Vosuga, and Sam Work. Dyer was in prison during the coup year, 1987, for his alleged involvement in an after-hours jewellery store robbery. By the late 1980s, he was back with Nadi, but he perceived that the administrators held something of a grudge against him because he had played for Lautoka. Hence his relations with the administrators were not as strong as before. After retirement, he became a coach of the village- and Indigenous-based Sweats Soccer Club, which played for a few years in the Nadi club competition.^4^

### Retirement transition

Dyer remarks as follows about the attitude of the Nadi Soccer Association administrators towards its retired stars around the time of his retirement:

1. Most of us soccer players who had played together for Nadi in the same era just completely dropped out of soccer commitment. It was like a guillotine or an axe on our shoulder or on our back. I can't remember any of us being active with the Nadi Soccer Association after that. We just completely dropped out and lost contact with each other. We did not go to watch Nadi games unless there was a tournament here in Nadi and there was an invitation. We had the feeling that we had done our part and that was it. It might be mentioned by the Nadi Soccer Association that there were coaching training clinics but there were no personal follow-ups. We all just forgot about soccer until we formed the Nadi Legends Football Club in 2004. This brought us together again [(32), p. 87, based on interviews conducted in 2014–2015].

Our interpretation is that it is the effective “banishment” to the Indigenous villages by the Association that is unnerving here–the ex-players have alienation forced upon them as they are no longer contacted or invited to fill in or apply for coaching roles. Things have improved somewhat since then at both district and FFA (league) level. The situation back then was especially uncaring as Nadi is a town of only 40,000 people and back then probably even fewer. Hence, staying in contact at the very least would not have been a difficult thing to do especially as many if not most ex-players lived in either Nakavu Village or Namotomoto Village, both of which are just over the Nadi River bridge on the opposite side to the town. By 2014–2015, Dyer was assistant village headman at Nakavu Village (he is the headman as the date of writing) but was banned from drinking in Farmers' Club (Nadi) and the Nadi Club when he should have been venerated and respected as an ex-Nadi star. However, he was welcomed at Lautoka pubs such as the Lautoka Club, the South Seas Club, and Renee's, because he had also been a Lautoka Blues icon.

Our statement “banishment to the Indigenous villages” does not mean to imply that the ex-players resent being in the villages or do not value their often senior and significant roles in village ceremonies and activities. The opposite is nearly always the case. However, they get disappointed when their soccer successes are forgotten and ignored by the administrators, the media, and younger fans. But they appreciate and enjoy the support and respect offered to them on a nearly daily basis by the hardcore fans of the 1980s teams including taxi-drivers and shopkeepers. Dyer receives more fundraising money than other villagers due to his soccer star status, charisma, and social skills, working as a package, but at that time in the mid-2010s no financial support was forthcoming from district or league administrators. In fact, in October 2015, the late Julie Sami of Ba revealed to me that he only received free admission to home IDC games and not to away IDC games or Premier League home-and-away clashes. Dyer explained as follows about his commitment to customary village obligations after retirement:

2. After playing active soccer, I became very committed to the community village life and to the protocols. While doing the Fijian or the *iTaukei* traditional and customary rituals of life, within the Nadi district, one has to be very tough at heart to sit down and to absorb all the talk and advice associated with observing the village hierarchy and relationships. You have to show the other side that you know your culture and the protocol. Whatever is going on today is a repetition of thousands of years back during the tribal wars.
  Therefore, to be committed to village life is not an easy thing. The good thing about village community life is that you have a lot of family around you and, when you are in need, your family is there to help. Even though most villagers are not well off we still survive. And our traditional relationships are not only here in the Nadi district but they are all around Viti Levu and Vanua Levu and the neighbouring smaller islands. So to live in the village is a mixture of the old past and the new present [(32), pp. 87–88, based on interviews conducted in 2014–2015].

Dyer talks about the self-help organization, Nadi Legends Club, aka Nadi Legends Football Club, established in 2004. This club was set up by ex-Nadi players and ex-officials, while the ex-official Mahend Singh is credited as being the founder. Unlike the earlier Sweats Soccer Club, it does not field teams in junior, senior, or women's leagues. It was a shining light for player self-help and even two Lautoka players, Jerry Ladawa and Joeli “Polo” Tora, joined and would make the trip down the Queen's Highway to Nadi on a regular basis [(32), p. 100]. At that point Lautoka Blues had no equivalent organization or at least not one of similar size and energy.

The aim of the club is to support ex-players in times of sickness and bereavement. A group of ex-players would plan a visit, hire a minibus if necessary, and go visit the sick ex-player. They can offer support and encouragement in a grounded and culturally appropriate way that does not involve outside paid experts or other prohibitive costs. In our view, in a money-poor but time-rich society, these sorts of initiatives should be praised. As of 2015, the Nadi Legends Club was the most advanced and well-known self-help club and the only one based around ex-players from the 1970s and 1980s rather than younger ex-players [(32), p. 100]. The following Henry Dyer quote describes the mission and vision of Nadi Legends Club:

3. Nadi Legends Club is really trying to show to the soccer fans and to other former district players that we should always look after each other on and off the field. When someone is falling down we should pull him up. As one of the songs says you can't keep a good man down. Why? Because there are people such as the Legends Club there to pull him up! One reason we have been together as a group and helping each other is that it is a culture and tradition which we are used to in the villages. We were brought up in this way since childhood so it is nothing new for us. The focus just changes from the family or the village to the soccer club. I am glad that our forefathers brought us up this way–to look after each other. If the Nadi Legends Club is funded or financed by some corporate body I believe that it can reach another level again from where it is now. Mahend Singh, who was instrumental in the formation of the club, and the rest of us are committed to the vision of remaining as a unified group with a common purpose whose members look after each other. The next step forward might be to have a physical meeting place or headquarters. At the moment the veterans' club trains at the Nadi Muslim College, which is good because it is central for everybody [(32), p. 101].

Dyer's next quote gives an example of a visit to the sick player Marika:

4. Marika [Vuniyawayawa] was a veteran full-back for Nadi. He played many years before my playing career. We visited him two weeks before he passed away at his home. He had some kind of sickness in the throat and he could not speak well. However, at our appearance, he sat up just to meet us. He could hardly talk but he was talking with the fire from inside. This shows you how a veteran feels when he sees another colleague or mate coming to visit him in his time of need. As old friends we told him to go and have his rest as we could see that he was struggling to talk to us. He said that he would not go to rest until our group had left his house, so we had to get away quickly! We had to leave without him knowing that we had gone so that he could have his rest. We had been doing this in the spirit of looking after each other during good times and bad times [(32), pp. 99–100].

In 2014, the FFA made its first tentative steps to care for ex-players' welfare by providing them with a structured opportunity for social interaction. However, as our primary data, including quotes from ex-Ba and Fiji striker, Inia “Golden Boot” Bola, indicates, this social function had several teething problems and weaknesses evident with hindsight. But before this, we should mention the Jone Nakosia Veterans' Tournament, which was a tournament involving four or five district teams and was designed to raise funds for the family of the deceased ex-Ba star Jone Nakosia. This tournament remains special and important because it was organized and funded by the ex-players. “Fiji Football felt that they had lost out on a marketing opportunity”, Dyer said to me in 2014 [(32), p. 98]. Shortly after this, the FFA got started on its efforts.

The following account draws upon participant-observation. The FFA organized and financed a Veterans' Dinner held in Nadi in October 2014 to coincide with that season's IDC tournament also held in Nadi.^5^ There was also a Masters IDC tournament in which the veterans were invited to participate, and this included teams representing both the Fiji districts and overseas regions. The Veterans' Dinner was held in the town council building on Nadi main street. Dyer and I shared Fiji Bitter long-neck beer bottles with a group of ex-players in the park adjacent to the town-council building as the sun was setting. Once we were inside, at a certain point, a young Fiji Indian man announced that only ex-players should be present, and I was personally told to leave. Dyer then went to talk with FFA President Rajesh Patel who, at that stage, was standing up at the front of the room and getting ready to speak. According to Dyer, Prasad backed down when confronted. In fact, Dyer's resolute and irrepressible cheerfulness, combined with his sheer determination to get his demands attended to, had won the day and I was permitted to stay.

After a short speech, the rows of chairs were reassembled in informal clusters spread around the room. Each ex-player took his white cardboard packet of hot noodles and Fiji Gold stubby bottle (375 mL) back to his group. Mostly the ex-players stayed with their own former teammates. The Ba contingent, including Lote Delai, stood and sat near the far wall and few people went over to interact with them. When I asked Dyer about this, he said that the older Ba players were not in attendance and only some younger players from a later era of Fiji soccer history had made the 60-kilometre trip. Nobody interacted with them, as except for Fiji national team player Lote Delai, the Ba players were unknown to the assembled Nadi contingent. This was the reason and not personal animosity.

At our 17 June 2015 interview with Inia “Golden Boot” Bola, who was mentally and physically damaged by the 1984 motor-vehicle accident on the King's Highway which killed Tubuna, Bola spoke incredulously and with some bitterness about the 2014 Veterans' Dinner. He complained that, because of his poverty and mental and physical condition, he could not go on his own steam and pay his own way to the Veterans' Dinner located 60 km away. Western readers may fail to grasp the point here, but Bola lived in the countryside at the back of Ba town, and bus travel there might have taken the whole afternoon. Furthermore, there would have been no chance of getting public transport back to his home late at night, as Ba basically shuts down after 5 p.m. apart from the occasional long-haul buses travelling to or from Suva. Taxis would have been prohibitively expensive for someone in his financial situation. “Do they think I am a taxi-driver?” He asked rhetorically at our interview. The following conversation, although clearly having an element of banter, as nearly all soccer-related conversations in Fiji do, remains depressing, albeit somewhat entertaining, reading ten years on:

5. Inia Bola: They come and ask me to the party. Do they think I am a taxi-driver?

Researcher: Do you mean the 2014 Veterans' Dinner in Nadi?

Inia: Yes. They just gave me a ticket to the dinner but no transport. We live far away and many of us don't work; we could not make it.

Researcher: The officials don't understand the way of life and the problems faced by the Fijians if they just give them tickets but don't assist them to go to the dinner.

Henry Dyer: The officials today do not think about how far inland people are or whether they are bedridden, crippled or not working. They don't find out how the guys are. They just pass over the tickets.

Inia: My ticket was passed to me by Semi Tabaiwalu. Rajesh Patel [FFA President] did not even arrange transport for us. It would have been great for the Ba boys from my era to have been at that function. I hope that this [situation] will not happen again (33).

According to our interpretation, lack of harmony between Bola's real situation and the FFA's lack of special arrangements for him is an illustration of Frankfurt School philosopher Theodor Adorno's (34) negative dialectics and the contradiction or gap existing between concept and object. The FFA, although knowing the reality very well, hid behind a kind of neoliberal “professional” “corporate” approach that is far better suited to and typical of a Global North context. Thus, like Pontius Pilate, they could wash their hands of the matter by pretending that the “corporate” way was the only natural, logical, or professional way, which is surely an ideological stance. There also remains mystery about who was invited to the dinner and who was not and, if some people were not invited, what were the reasons, if any. At the actual dinner, there was no ex-Ba player present from Bola's era and as a result mixing between teams was nonexistent–Meli Vuilabasa was not there nor was Julie Sami, Bola, or Semi Tabaiwalu.

### Cultural identity

We now move on to the case of ex-Ba and Fiji player, Semi Tabaiwalu, and the fact that his coaching contract was not renewed after four years by Ba Football Association. Tabaiwalu was a member of Ba's six-in-a-row IDC title winning side from 1975 to 1980 that brought great pride and joy to this small, provincial market town located in the northwest of Viti Levu 30 kilometres up the coast from Lautoka. Stories were told by our Ba interviewees and their wives of the appreciation towards the players shown by townsfolk and shopkeepers (35). There were parties at the Ba River foreshore that lasted for days on end with people coming and going while the party continued. There are stories of shopkeepers allowing players free clothes and other goods from the shops and then allowing their wives to come down to the same shops later in the day to receive further goods (35). These stories may seem quaint or trivial by Global North standards, but it should be remembered that the game was basically amateur even in the Fiji Premier League back then. The gifts came under the heading of benevolent paternalism, as do the parties, as it is unlikely that the mostly Indigenous players bought any drinks. As the administrators and shopkeepers were mostly Fiji Indians, the benevolent paternalism had an ethnic aspect or operated along ethnic lines. We say “paternalism” as those richer administrators and fans would have chipped in more cash for drinks and gifts, and would, according to cultural norms, have been expected to do so.

Tabaiwalu was appointed head coach of the Ba Football Association senior team in the Fiji Premier League (“manager” in the parlance of English and European soccer) and his time in charge would be declared successful by anybody's criteria with each one of the four annual trophies having been won at least once between 2007 and 2010 [(30), pp. 198–216]. Tabaiwalu remains somewhat aggrieved, or he was at our interview date of 20 June 2015, that his contract was not renewed after his four-year term ended. He claims that the reason was that he was too honest, direct, and outspoken and the administration wanted a more pliable coach.

In the Indigenous kava ceremony, held at village functions, there is a strict order of hierarchy and seniority with the most important person present being served first, followed by the second-most important person, and so on [(36), p. 154; (37), p. 44]. The kava bowl must be handed to the drinker by the server without the bowl touching the ground and the same applies with the return of the bowl. To spill some on the floor is seen as partaking with evil spirits and is unacceptable. People must be seated on the floor, and no part of the legs or feet may be raised above the floor. The standard way of sitting is the cross-legged position [(37), p. 52]. The drinking of beer often mirrors the kava ceremony [(37), p. 61] in the way that the server pours beer into a small glass (*bilo*) and serves (*taki*) it direct to the drinker [(38), p. 103]. Some aspects of the kava ceremony still operate, such as solidarity and respect for hierarchy and order [(37), pp. 62, 76], but there is an element of cheerful subversion that almost but not quite becomes parody [(38), pp. 100–101]. Glasses are often handed from drinker to drinker [(37), p. 76] and the person buying the alcohol may be served first or second whereas in the kava ceremony strict hierarchy order is never violated [(36), p. 161; (37), pp. 61, 76; (38), p. 104]. A more jovial atmosphere, rather than a solemn one, tends to prevail.

The following account draws upon participant-observation. The Ba Central Club is a two-storey, membership-based club located at the top of a hill in Ba Town Centre with awesome views of rolling hills and farmland out the back. It is similar to Lautoka Club and Nadi Club but struggles for customers due to Ba's small size. It is most probably closed at the date of writing. It was known as a bastion for the Ba Football Association administrators including Rajesh Patel, then FFA President. On Saturday, 20 June 2015, Tabaiwalu was nervous but inwardly defiant as we entered the Ba Central Club. We wanted him to be able to hold his head up high again after his dismissal from Ba which was then only four years in the past. We started talking in jovial manner with the Fiji Indian bar staff who knew who Dyer and Tabaiwalu were. In Ba they love a soccer conversation at any time of day or night and any day of the week so there was no problem with the atmosphere at least as far as I could tell. Dyer poured some Fiji Gold from a longneck into a glass and offered it to one of the barmen, kava ceremony style. This was an existential test as to drink it would signify fellowship and solidarity. To everyone's delight the barman took the drink and downed it and I have the picture that was taken at just the right second from my camera. This day was an interview day, a participant-observation day, but also the day when we hoped Tabaiwalu would get his self-esteem back.

Tabaiwalu, now aged 72, was invited in November 2024 to Sydney by the Fiji Indian Diaspora there to speak at a special Fiji soccer night along with several other ex-stars (including Meli Vuilabasa, Inia Bola, Rupeni Soro, Satish Kumar, and the late Julie Sami) (39, 40). For years Tabaiwalu was invited there to coach the “Ba team” in replica IDCs held among the émigrés, with nearly all players being émigrés or sons or nephews of émigrés. This honour was something of a consolation in his mind for his earlier rejection by the official Ba powerbrokers. However, Tabaiwalu told us he would not want to live in Australia or New Zealand as he would miss the Indigenous Fijian cultural way of dropping in at village homes and staying for dinner and sleeping the night.

### Institutional responses

Lastly, we look at more recent FFA initiatives in terms of helping the families of ex-players (termed veterans). During the awards ceremony of the RC Manubhai/Apco Coatings Veterans' Tournament held at the HFC Bank Stadium in Suva, the FFA presented cheques for FJD500 to the families of four veterans, Nadi rep Dan Lutumailagi, Suva striker Tony Kabakoro, Rewa goalkeeper Vula Wate, and Nadi striker Rusiate “Waqan” Waqa (41). The Veterans’ Tournament revenue made the payments possible, and the money went towards helping fund children's and grandchildren's education. “We believe in honoring those who paved the way for today's generation. These players gave their all for the nation, and this is our way of saying they are not forgotten”, said the FFA's Rajesh Patel. “As the Veterans Tournament grows, we hope to increase the number of families supported. Football is more than a game—it's a community, and we stand by those who gave so much to it.” The Trustees of the Veterans' Committee chose the recipients. This financial scholarship is a commendable gesture in a country with minimal welfare benefits. However, ex-players without school-aged children or grandchildren clearly will not be able to qualify for a benefit nor obviously will those ex-players not chosen for the scheme. Our interpretation here is that the scheme is certainly a very tailored scheme, backed up by conservative family values ideology, which is not to say that the scheme should not happen. In an earlier development, around 2017–2019, Inia Bola was named as one of the inaugural FFA Legends of the sport.

### About benevolent paternalism and cultural hegemony

Although Foucault described conditions within modernist institutions in France after 1790, the power/knowledge concept can be applied to Fiji and Fiji soccer with the caveat that power/knowledge works by and through traditional methods of control, such as benevolent paternalism and cultural hegemony, as well as modern methods. While the long-term friendship between Dyer and Tikaram extends to both professional and personal spheres, such relationships do not always exist or are not always so beneficial. Tikaram is a special individual in terms of his compassion and man-management skills. We see Bola and Tabaiwalu having more troublesome experiences. Tabaiwalu was managed by Indian cultural hegemony, and he was perceived, according to him, as being too direct and outspoken as a coach. He was nearly certainly managed and judged by the Indian cultural cues and expectations of cultural hegemony, but being of a different culture and religion, he may have misread or resented the cues and signals. Dyer never held a senior coaching job at district or national-team level and his jail time for a jewellery store robbery in 1987 probably was held against him given that the soccer administrators were mostly businessowners. Despite this, the lack of a pathway for Indigenous coaches and administrators surely adversely affects the health of the sport. There are some parallels here with the white management/black players situations in the UK and US.

## Conclusion

It is not easy to relate the Fiji primary data to the earlier section on the European literature. The cultures are very different as are, obviously, the levels of expertise and financial resources and infrastructure that can be tapped into. For example, in the typical Fiji town or city, there are unlikely to be any psychologists specializing only in sport and few or zero qualified and experienced nonspecialist psychologists. Slowly this will change. Resources and distance (remoteness) are a problem as is the constant drain of talented people to countries such as Australia, New Zealand, and Canada.

No matter what happens, an aping of Western methods will not be appropriate given the lack of resources, infrastructure, and knowledge on the ground. Traditional benevolent paternalism, as shown by Tikaram, should not be looked down upon during transitional times and the Nadi Legends Club is clearly ministering effectively (the religious term is used on purpose here) to ex-players in a culturally appropriate and grounded manner. Even should the country develop rapidly, Nadi Legends Club and similar groups will continue to play an important role as ex-player-helping-ex-player adds something special and irreplaceable to the mix.

The FFA has begun in recent times, beginning around 2014, to assist the ex-players and look after their welfare. However, the neoliberal approach of “hiding” behind corporate structure and corporate practices has meant that some practical needs have been unmet, such as Inia Bola's transport to and from the 2014 Veterans' Dinner. To answer Bola's question, posed to Dyer and me in June 2015, they knew he wasn't a taxi-driver! The hiding behind corporate logic and behaviours, whilst partly a response to hegemonic pressures from inside and outside the country, has meant that a strange kind of blindness, a blindness on purpose, has prevailed at times. But Rajesh Patel and his administrators cannot be blamed for events prior to their taking over FFA, as we have written elsewhere [(32), p. 98]. The FFA is now recognizing some type of moral obligation to ex-players, although the actual extent of its moral and legal obligations is uncertain even from a Global North view. Will clubs and/or leagues be held accountable for ex-players who go on to suffer dementia where this can be traced to heading the football during their careers? Hutcheon (10) cites a new Glasgow University report which suggests that in Scotland social security benefits could be given to ex-players with this condition.

We now highlight what lessons can be drawn for sports governance, coaching education, and athlete welfare in similar Global South contexts. In sum, a mix of modern/European and traditional support and care is recommended in the Fiji Islands and similar countries. It would be a mistake should anyone want to replace completely the latter with the former both because of cultural and ethical reasons, on the one hand, and the ongoing (lack of) resources and expertise, on the other.

A similar conclusion was reached by Gill Barber (42) in her anthropological study of traditional midwives in Malawi. Given the lack of financial resources and expertise in the country in relation to midwifery she supported use of traditional midwives since they were trusted and were better than women giving birth alone or with less experienced helpers. In a more developed Malawi, she would still want to see traditional midwives operating side-by-side with hospital staff trained and equipped in the modern way.

Fiji has declined in soccer standards, as indicated by global ranking in the game, since the 1980s, compared to neighbouring countries. Reasons are hard to pin down but might include lack of imagination from the administrations and the rise in popularity of 7 s and 15 s rugby and rugby league since the early 1990s. The rise in popularity of rugby is a factor that soccer administrators cannot be blamed for, although there are lessons from those other sports that can be learned. The media should put big soccer tournaments on the back pages of the newspapers, usually reserved for rugby, and the administrators should continue their efforts to expound an inspiring Fiji soccer narrative that draws upon the best events in soccer history and highlights that soccer is a national pastime as well as the World Game. Links should be formed in the region and beyond with associations and clubs overseas, and ex-star players should be decorated and not forgotten. In the past, players such as Kelemedi “Cheetah” Vosuga went to Brisbane City in Australia and, more recently, Esala Masi Junior played at Gippsland Falcons, Wollongong Wolves, and Newcastle United Jets, while in the 1980s Canberra's West Woden Juventus toured Fiji. Australia's departure from the Oceania conference to join the Asian conference of FIFA has probably had a long-term negative effect in Fiji by reducing matches between the two countries and hindering the formation of new links.

The contribution of businessmen as presidents and sponsors should be continued, with newer ones replacing old, and they, in conjunction with government, can help modernize the sport including the infrastructure. We note the major ground improvements undertaken recently at Ba's Govind Park stadium.

Overall, we need in the Global South a mix of traditional and modern. Overseas visiting experts (and I was one of them) must not tend towards either the extreme of relaxing too much and enjoying fringe benefits in a conducive and beautiful setting or the other extreme of imposing Western policies wholesale without the accompanying money, culture, education, or infrastructure. Change should occur piece-by-piece in terms of introducing overseas policies and the cultural styles of the local cultures respected and worked with. New policies and technologies can be used and implemented and followed *in the cultural style and way*, unless this translates into actual corruption or obvious inefficiencies. Foreign coaches and players should be allowed and encouraged, but with numbers capped. There needs to be a willingness to learn and change on behalf of locals, but this does not mean that key elements of cultures should be disrespected or disregarded. *Leaders must get people onboard*. My Fiji experience was that, due to lack of policies and rules, change can occur quickly if people agree. If they do not agree, nothing will change for 100 years.

## Data Availability

The raw data supporting the conclusions of this article will be made available by the authors without undue reservation.

## Footnotes

1 As at the 2007 Census, Indigenous Fijians made up 56.8% of the population of Fiji, while Fiji Indians (Fijians of Indian descent) made up 37.5%.

2 Prasad [(30), p. 108] gives the score as 8-1 but alternative (rumoured) scores are 8-0 and 7-1.

3 Dyer joined just after Lautoka's 1985 IDC win against Rewa in Ba [(32), p. 62].

4 The Sweats Soccer Club story has been detailed elsewhere [(32), Chapter 6, pp. 87-93] and won't be recounted here.

5 “Veterans” in this context means ex-national-league players and not former soldiers.
